# Alternative Definitions of Floral Resource Availability Alter Inferred Plant Importance in Plant‐Pollinator Networks

**DOI:** 10.1002/ece3.73877

**Published:** 2026-06-23

**Authors:** Han Yan, Ian P. Vaughan, Jane Memmott

**Affiliations:** ^1^ School of Biological Sciences, University of Bristol Bristol UK; ^2^ School of Biosciences, Cardiff University Cardiff UK

**Keywords:** floral resource availability, keystone species, methodological uncertainty, null models, plant‐pollinator networks

## Abstract

Null models are widely used to infer pollinator preferences from plant‐pollinator visitation networks, but these inferences depend on how floral resource availability is defined. This is particularly important for plants with complex floral structures, where individual flowers, floral units and larger inflorescences may each represent biologically plausible resource units. Here, we used *Heracleum sphondylium* (common hogweed), an Apiaceae species with hierarchical umbels, to test whether alternative definitions of floral resource availability alter null model inference in plant‐pollinator networks. Plant‐pollinator interaction data were collected from three field sites near Bristol, UK (Brandon Hill, Fenswood Farm and Leigh Woods), and hogweed floral abundance was quantified using three definitions: individual flowers, floral units and umbels. We found that the inferred importance of hogweed depended on the definition of floral resource availability. Hogweed was generally classified as a potential keystone plant when abundance was defined using floral units or umbels, whereas individual flower counts produced more variable classifications. Changing the resource definition of hogweed also altered the inferred importance of other plant species, with 12% to 39% of plant species × insect order classifications differing among resource definitions across sites. Broader inferred pollinator preference patterns also differed among resource definitions at some sites, although the magnitude of this effect varied among communities. These results show that floral resource definitions can influence ecological inference from the same observed visitation data. Explicitly defining and reporting floral resource units is therefore essential when using plant‐pollinator networks to infer pollinator preferences and plant importance.

## Graphical abstract

This study shows that alternative definitions of floral resource availability can alter the outcomes of pollinator preference null models, affecting both potential keystone plant identification and broader network interpretation. Using *Heracleum sphondylium* as a case study, we demonstrate that methodological choices in floral abundance quantification can influence ecological inference even when the underlying visitation data remain unchanged.

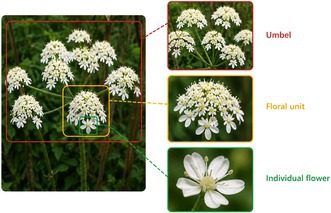

## 1 Introduction

Insufficient floral resource availability has been identified as an important contributor to pollinator declines (Potts et al. 2010). Identifying which plant species provide important resources for pollinators is a central question in community ecology and conservation. Within plant‐pollinator interaction communities, the ecological importance of a flowering plant is commonly evaluated through how its floral resources are used by flower visiting insects and contribute to their foraging, survival and reproduction. However, directly measuring these contributions across entire communities is challenging. As a result, plant–pollinator networks are widely used as a practical framework for assessing patterns of resource use (Biella et al. 2020; Zoller et al. 2023) and identifying plant species that may play disproportionately important roles within communities (Pocock et al. 2012).

Within pollination networks, some plant species are visited more frequently by certain pollinator groups than by others, reflecting variation in foraging behaviour and floral preferences. Null models could be used to evaluate these visitation preferences by comparing observed visitation frequencies with those expected based on floral resource availability (Vaughan et al. 2017). This comparison allows researchers to assess whether insect pollinators visit particular plant species more or less frequently than expected relative to their floral abundance, and to identify species that receive disproportionately high visitation. In this study, we refer to these species as potential keystone plants (Whittaker and Cottee‐Jones 2012).

A critical but often overlooked assumption of these approaches is how floral resource availability is defined and quantified. Floral abundance is not merely a descriptive variable, but determines the null expectation against which observed visitation is compared. Measuring floral abundance is not always straightforward because flowering plants differ greatly in floral architecture, display size and inflorescence structure (Michelot‐Antalik et al. 2025). Individual flowers, functional floral units, and larger inflorescences may each represent biologically plausible measures of resource availability, depending on floral morphology and how insects perceive and use floral resources. Consequently, alternative definitions of floral abundance may alter the expected patterns of resource use in preference‐based null models, leading to different interpretations of pollinator preferences and plant importance within communities.

Plants with hierarchical inflorescences are widely represented in pollination networks, including many species in Apiaceae and Asteraceae. *Heracleum sphondylium* (common hogweed, hereafter hogweed), a member of the Apiaceae, provides a suitable system for examining how alternative definitions of floral resource availability influence ecological inference in plant‐pollinator networks. Hogweed has a complex hierarchical floral structure, with numerous small individual flowers grouped into floral units, which are further arranged into larger umbels (Zych 2007). These nested floral structures allow its abundance to be quantified at several biologically plausible scales. Hogweed is also an ecologically relevant focal species: it is native to most of Europe (Senejoux et al. 2013); flowers through much of the summer in southern Britain, overlapping with our sampling period; and it is visited by a wide range of insects, including bees, beetles and flies (Balfour et al. 2022). Previous studies have identified hogweed as a potential keystone plant species based on consistent visitation preferences expressed by multiple pollinator taxa across habitats (Pocock et al. 2012; Baldock et al. 2019).

**FIGURE 1:**
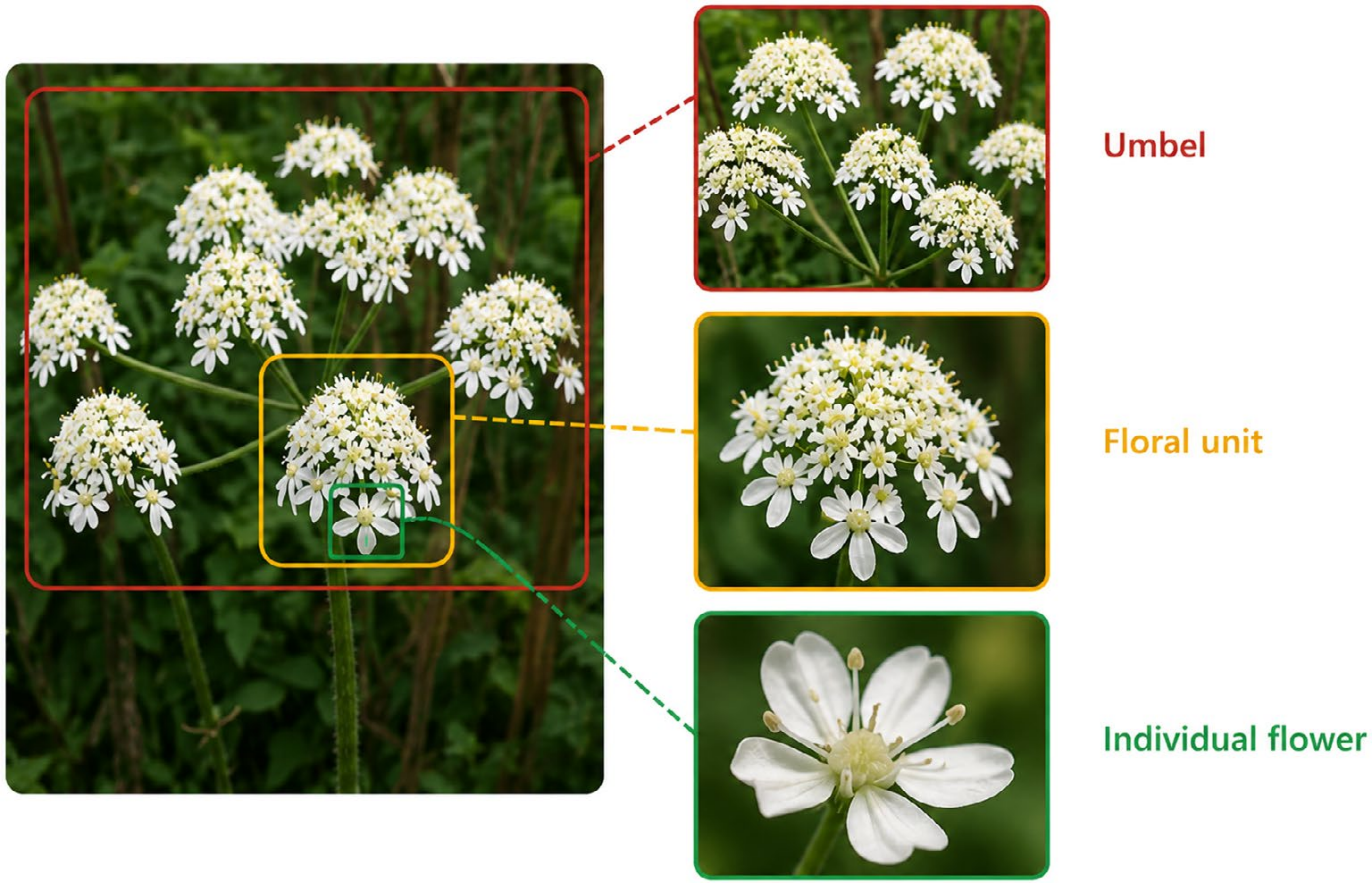
Floral resource definitions used for *Heracleum sphondylium* (common hogweed). Three hierarchical counting scales were compared: individual flowers, floral units (clusters of individual flowers on a pedicel), and umbels (groups of units on the same stem or peduncle). These alternative definitions represent different quantifications of floral resource availability for the same focal species.

We quantified hogweed floral abundance using three alternative definitions of resource availability: individual flowers, floral units, and umbels (Figure 1). In this species, floral units are clusters of flowers borne on a pedicel, whereas umbels are groups of floral units borne on the same stem or peduncle. Using preference‐based null models, we tested whether alternative definitions of floral resource availability influence ecological inference in plant‐pollinator networks. The study had three objectives. First, we assessed whether the inferred importance of hogweed as a potential keystone plant depends on how floral resource availability is defined. Second, we evaluated whether changing the resource definition of a focal plant species influences the inferred importance of other plant species within the community. Third, we examined whether alternative definitions of floral resource availability alter broader patterns of inferred pollinator preference across the network.

## 2 Methods

### 2.1 Field Sites

Three field sites in or near Bristol, UK, were selected based on their suitability for repeated plant‐pollinator visitation observations and floral abundance surveys: Brandon Hill (51°27′N 2°36′W), Fenswood Farm (51°25′N 2°40′W) and Leigh Woods (51°28′N 2°39′W). Sites selection considered both ecological and logistical criteria: ecologically, each site supported flowering hogweed during the sampling period, and included a wider flowering plant community suitable for constructing plant‐pollinator visitation networks; logistically, sites were required to be accessible for repeated weekly sampling, large enough to allow repeated observations, and to contain open, sunny sampling areas where insect visitation could be observed under suitable weather conditions.

The three sites also represented contrasting habitat contexts. Brandon Hill is an urban nature reserve with open meadow vegetation, Fenswood Farm is a working farm with open meadow areas, and Leigh Woods is a woodland site where sampling was conducted in a herbaceous plant community alongside a track. At each site, one plot of approximately 1500 m^2^ was established and used throughout the study. The three study plots were therefore comparable in sampled area, although they differed in habitat context.

### 2.2 Data Collection

Field observations were conducted from early June to early September, coinciding with the peak flowering period for hogweed in this region (see the Supporting Information of Timberlake et al. 2019). Each field site was sampled once a week on sunny days when the temperature was above 16°C and wind speed was no more than moderate, to ensure suitable conditions for insect activity and standardise sampling conditions. Insect visitation was recorded using timed walking surveys. During each survey, the observer walked through the plot at a constant pace and recorded all insects landing on open flowers. Each insect was captured with an insect net, transferred to a labelled tube with a unique specimen number, and the plant species visited was recorded. The timer was paused during capture and labelling, so that handling time was excluded from the observation period. Insects were subsequently identified to species by a professional entomologist (see acknowledgements). One 45‐min survey was conducted for the first three sampling visits at each site; thereafter, two 45‐min surveys were conducted per visit to increase the sample size under conditions of low seasonal insect density. Due to premature agricultural management (mowing) at Brandon Hill, sampling at this site was restricted to seven visits, whereas Fenswood Farm and Leigh Woods were sampled 13 and 12 times, respectively.

Following each insect visitation survey, floral abundance was recorded at each site using 1 m × 1 m quadrats. Six equally spaced 30 m transects were established within each plot, with seven quadrats placed along each transect at 5 m intervals. All flowering plant species within each quadrat were identified and their floral abundance recorded. The abundance of hogweed was quantified using three alternative definitions of floral resource availability: individual flowers, floral units and umbels (Figure 1); all other plant species were quantified following established protocols using floral units, defined as individual flowers or clusters of flowers within which an insect of approximately 0.5 cm could walk within or fly between (Gibson et al. 2006; Power and Stout 2011). The floral abundance for species with very small and numerous flowers, e.g., *Galium* species (bedstraws), was estimated by subsampling the number of individual flowers and multiplying up. The visitation data and floral abundance data were collected on the same sampling day by the same observer throughout the field season.

### 2.3 Null Model Analysis

Keystone identification was based on preference‐based null models implemented using the R package *econullnetr* (Vaughan et al. 2017). Following previous applications of this approach and to reduce sparsity in species‐level interactions, visitation data were aggregated to insect order level. For each plant species × insect order combination, observed visitation frequencies were compared with expected frequencies generated by the null model, using 95% confidence intervals derived from 20,000 iterations. Standardised effect size (SES) was calculated as the difference between observed and expected visitation frequency, divided by the standard deviation of visitation frequency across the 20,000 null model iterations. Interaction were classified as stronger than expected (SES > 2), weaker than expected (SES < −2), or not significantly different from null expectations (−2 ≤ SES ≤ 2). In this null model context, positive SES values indicate more visits than expected based on floral resource availability, and negative values indicate fewer visits than expected. Plant level inference was then summarised from these interaction level results. A plant species was classified as a potential keystone plant within a network if it showed one or more significantly stronger than expected interactions with any insect order, indicating that it received disproportionately high visitation relative to its floral availability. All analyses were performed using R version 4.0.2.

### 2.4 Objective 1: Effects of Floral Resource Definitions on the Inferred Importance of Hogweed

For each field site, plant‐pollinator visitation data collected across all sampling occasions were combined to construct a single site‐level network representing the full sampling period. Each site‐level network was analysed three times using the null model, with identical visitation data but with hogweed floral abundance quantified using individual flowers, floral units or umbels. This generated three sets of null model outputs for each site, allowing the inferred interaction strength and potential keystone classification of hogweed for each insect order to be compared across alternative definitions of floral resource availability. We then compared these classifications across the three floral resource definitions to assess whether hogweed remained consistently classified as a potential keystone plant or changed status depending on how its floral abundance was defined.

### 2.5 Objective 2: Effects of Hogweed Resource Definitions on Inferred Importance of Other Plant Species

Using the same null model outputs generated for Objective 1, we evaluated whether changing the floral resource definition of hogweed influenced the inferred importance of other plant species within each community. Because the observed visitation data were identical across the three model runs within each site, any differences in inferred interaction strength or classification for non‐focal plant species could be attributed to differences in the floral abundance definition of hogweed.

For each site, we compared the interaction classification of each non‐focal plant species × insect order combination across the three hogweed resource definitions. Interactions were classified as significantly stronger than expected, significantly weaker than expected, or not significantly different from null expectations, following the SES thresholds described above. A classification was considered consistent when the same plant species × insect order combination received the same status under all three hogweed resource definitions. We then calculated the percentage of non‐focal plant species × insect order combinations with consistent classifications. This measure captured the stability of null model inference across alternative definitions of hogweed floral abundance, including classifications that were consistently stronger than expected, consistently weaker than expected, or consistently not different from null expectations.

### 2.6 Objective 3: Effects of Floral Resource Definitions on Inferred Pollinator Preference Patterns

To assess whether alternative hogweed resource definitions affected broader patterns of inferred pollinator preference, we compared SES matrices generated from sampling occasion level networks. Unlike Objectives 1 and 2, which used site‐level networks pooled across the full sampling period, this analysis used replicate networks constructed from individual sampling occasions. Sampling occasions in which hogweed was absent from the floral abundance transects were excluded from this analysis, because hogweed abundance could not be redefined across the three resource definitions on those occasions. This left three replicate networks from Brandon Hill, seven from Fenswood Farm, and eight from Leigh Woods. The small number of replicate networks at Brandon Hill was due to early mowing at this site.

For each remaining sampling occasion, the same visitation data were analysed three times using the null model, with hogweed floral abundance quantified as individual flowers, floral units or umbels. This produced three SES matrices for each replicate network, one for each hogweed resource definition. Each SES matrix contained plant species × insect order interaction values, representing the extent to which observed visitation was stronger or weaker than expected given floral resource availability.

We then calculated Euclidean distances among SES matrices within each site and used non‐metric multidimensional scaling (NMDS), implemented in the R package *vegan*, to visualise differences in inferred pollinator preference patterns among hogweed resource definitions (Oksanen et al. 2022). Euclidean distance was used because SES values include both positive and negative deviations from null expectations, making abundance‐based dissimilarity measures such as Bray Curtis inappropriate. Differences in inferred preference patterns among floral resource definitions were tested using permutational multivariate analysis of variance (PERMANOVA; *vegan:adonis2*), with 999 permutations.

## 3 Results

Sampling from the three field sites recorded the interactions among 217 species of insects (2280 individuals) and the flowers of 32 flowering plant species. The insects were 975 Hymenoptera (43% of insects; 63 species), 870 Diptera (38% of insects; 110 species), 346 Coleoptera (15% of insects; 15 species), 20 Lepidoptera (< 1% of insects; 11 species) and 69 Hemiptera (3% of insects; 18 species).

### 3.1 Objective 1: Effects of Floral Resource Definitions on the Inferred Importance of Hogweed

**FIGURE 2:**
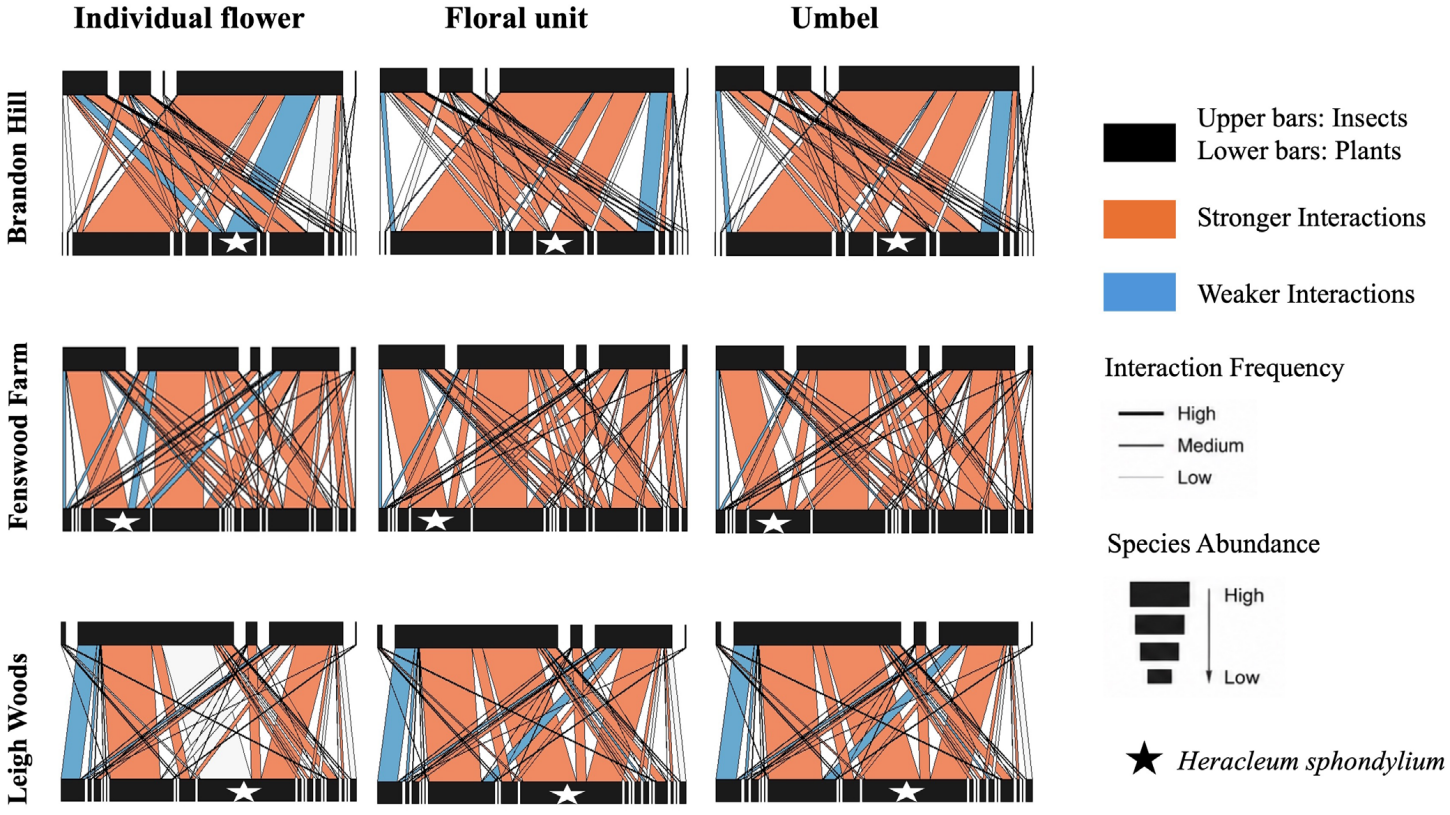
Plant‐pollinator visitation networks across field sites and hogweed floral resource definitions. Plant‐pollinator visitation networks for the three field sites under three alternative definitions of hogweed floral resource availability: Individual flowers, floral units and umbels. Panels show networks for a meadow site, Brandon Hill; a farmland site, Fenswood Farm; and a woodland site, Leigh Woods. The upper bars represent the relative abundance of insect orders, including Coleoptera, Diptera, Hemiptera, Hymenoptera and Lepidoptera respectively. The lower bars represent the surveyed floral abundance of plant species. Link width indicates the observed frequency of interactions between insect orders and plant species. Red links indicate interactions that were significantly stronger than expected under the null model (which are the potential keystone plants), blue links indicate interactions that were significantly weaker than expected, and white links indicate interactions that were not significantly different from null expectations. Very thin white links may appear black because of their borders. The bar represent focal plant *Heracleum sphondylium* (hogweed) is marked with a white star.

**FIGURE 3:**
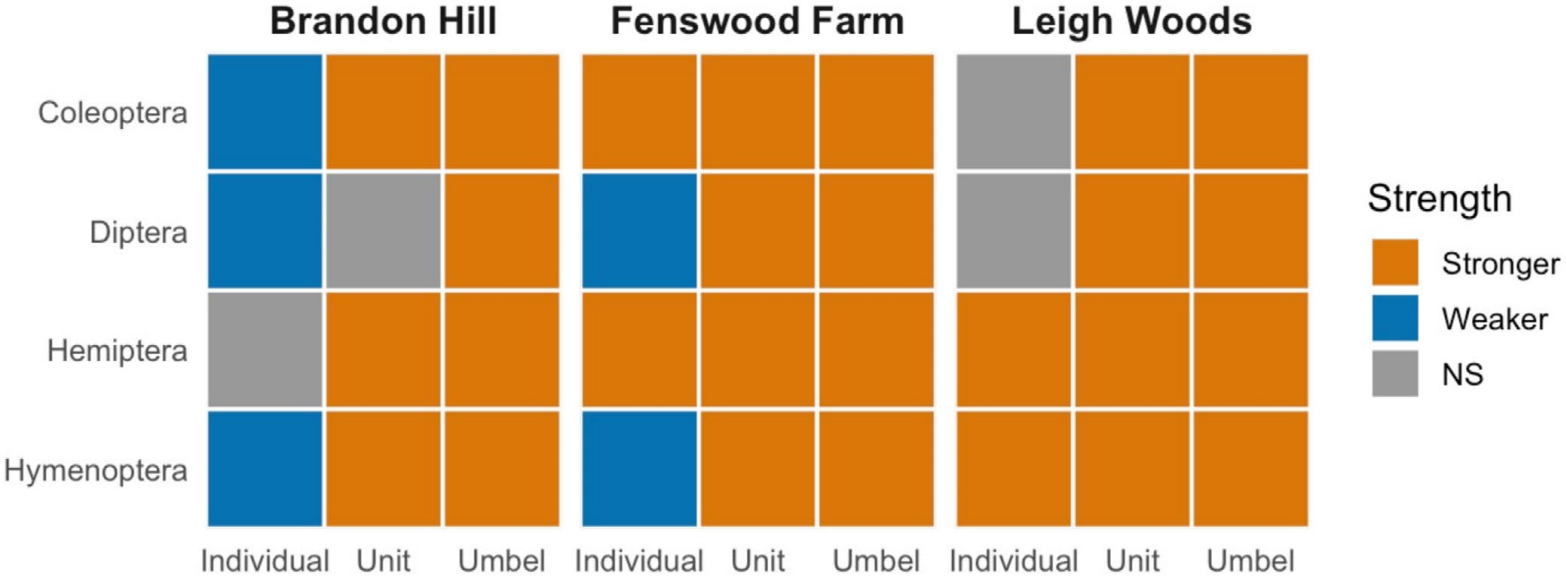
Inferred importance of hogweed across field sites and floral resource definitions. Null model classifications of interactions between hogweed and four insect orders across three field sites under three definitions of hogweed floral resource availability. Columns represent individual flowers, floral units and umbels, and rows represent insect orders. Tile colours indicate interactions that were significantly stronger than expected (potential keystone classification), significantly weaker than expected, or not significantly different from null expectations.

Plant‐pollinator networks for each field site were plotted with interactions classified according to null model outputs generated under the three hogweed floral resource definitions (Figure 2). The inferred importance of hogweed, assessed through its classification as a potential keystone plant, depended on how its floral resource availability was defined. For each field site and insect order, we compared the null model classification of hogweed under the three floral resource definitions: individual flowers, floral units and umbels (Figure 3). These comparisons showed that hogweed was not always consistently classified as a potential keystone plant across resource definitions. Floral unit and umbel definitions produced broadly similar classifications, with hogweed classified as potential keystone species for Hymenoptera, Hemiptera and Coleoptera across all three sites. In contrast, defining hogweed abundance using individual flowers often produced classifications that differed from those obtained using floral units or umbels. The clearest change occurred for Diptera at Brandon Hill, where hogweed was classified as significantly weaker than expected when abundance was defined using individual flowers, not significantly different from null expectations when defined using floral units, and significantly stronger than expected when defined using umbels. These results show that changing the floral resource definition can alter whether hogweed is inferred as a potential keystone plant, even when the observed visitation data are unchanged.

### 3.2 Objective 2: Effects of Hogweed Resource Definitions on Inferred Importance of Other Plant Species

**FIGURE 4:**
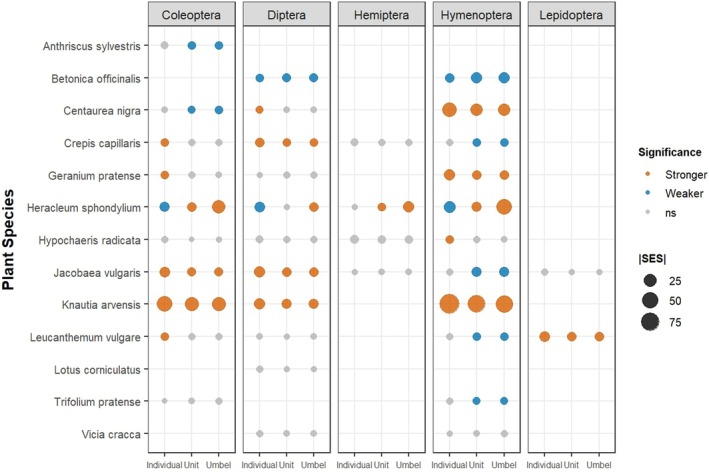
Null model classifications and SES values for plant species at Brandon Hill. Points show plant species × insect order interactions under three definitions of hogweed floral resource availability: individual flowers, floral units and umbels. Point colour indicates whether interactions were significantly stronger than expected, significantly weaker than expected, or not significantly different from null expectations. Point size represents the absolute SES value.

Changing the floral resource definition of hogweed also influenced the inferred importance of other plant species within the community, as reflected by changes in their null model classifications. Across the three field sites, the proportion of plant species × insect order classifications that differed among the three hogweed resource definitions ranged from 12% at Fenswood Farm and Leigh Woods to 39% at Brandon Hill. Brandon Hill was therefore used as a detailed example because it showed the greatest sensitivity to changes in hogweed resource definition (Figure 4). Some plant species showed consistent classifications across all three definitions. For example, *Knautia arvensis* was consistently classified as a potential keystone plant for Coleoptera, Diptera, and Hymenoptera regardless of how hogweed floral abundance was defined. In contrast, other species changed classification depending on the hogweed resource definition. For example, *Centaurea nigra* , *Crepis capillaris* , and *Geranium pratense* lost their potential keystone classification for some insect orders when hogweed abundance was defined using floral units or umbels rather than individual flowers. Changes in classification were also reflected in SES values, with hogweed SES values increasing and other plant species decreasing across the same resource definition sequence.

### 3.3 Objective 3: Effects of Floral Resource Definitions on Inferred Pollinator Preference Patterns

**FIGURE 5:**
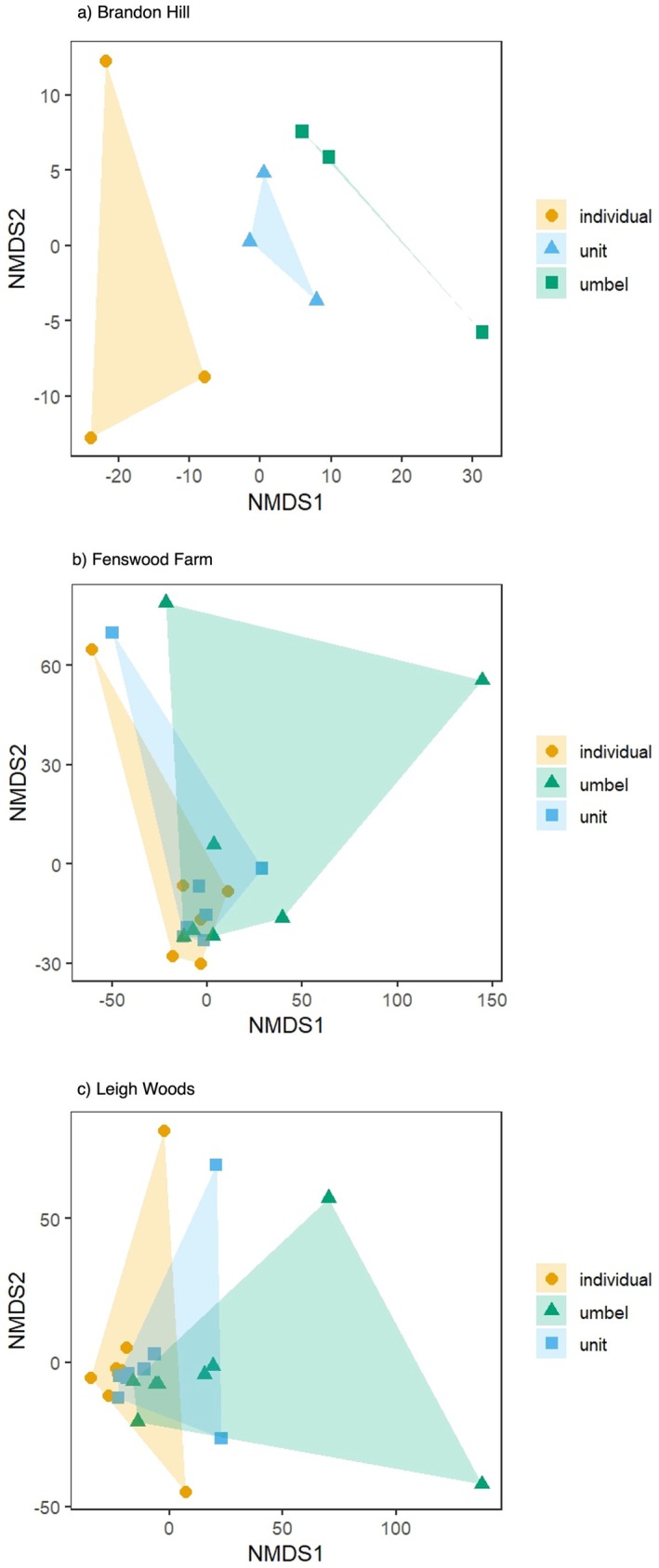
Differences in inferred pollinator preference patterns among hogweed floral resource definitions. NMDS ordination based on Euclidean distances calculated from plant species × insect order SES matrices generated by null model analyses. Points represent replicate sampling occasions, and colours represent the three definitions of hogweed floral resource availability: individual flowers, floral units and umbels. Polygons show the distribution of replicate networks within each resource definition.

Differences in inferred pollinator preference patterns among the three hogweed floral resource definitions were assessed using NMDS ordination of SES matrices (Figure 5) and PERMANOVA (Table 1). The effect of hogweed resource definition varied among field sites, indicating that changes in floral resource definition can influence broader null model inference, but not uniformly across communities. At Brandon Hill, inferred preference patterns differed significantly among the three hogweed floral resource definitions (PERMANOVA: *F* = 4.0445, *R* ^2^ = 0.574, *p* = 0.010). The NMDS ordination showed clear separation among SES matrices generated using individual flowers, floral units and umbels (Figure 5a). At Fenswood Farm, inferred preference patterns did not differ significantly among floral resource definitions (PERMANOVA: *F* = 1.2255, *R* ^2^ = 0.12, *p* = 0.255). This was consistent with the substantial overlap among groups in the NMDS ordination (Figure 5b). At Leigh Woods, floral resource definition had a significant but weaker effect on inferred preference patterns compared with Brandon Hill (PERMANOVA: *F* = 2.6622, *R* ^2^ = 0.202, *p* = 0.018). The NMDS ordination showed partial separation among floral resource definitions, although there was still considerable overlap among groups (Figure 5c). Overall, these results suggest that changing the floral resource definition of hogweed can affect inferred pollinator preference patterns beyond individual plant classifications, but that the magnitude of this effect depends on field site.

**TABLE 1:** PERMANOVA results testing differences in inferred pollinator preference patterns among hogweed floral resource definitions.

Statistical result/site	Brandon hill	Fenswood farm	Leigh woods
Degree of Freedom (Residual)	6	18	21
*R* ^2^	0.574	0.12	0.202
*F*	4.0445	1.2255	2.6622
Pr (>*F*)	0.01*	0.255	0.018*

*Note:* * *p* < 0.05.

## 4 Discussion

This study demonstrates that the way floral resource availability is quantified can influence ecological interpretations derived from plant‐pollinator network analyses. Using *Heracleum sphondylium* as a focal species, we showed that alternative definitions of floral abundance altered whether hogweed was classified as a potential keystone plant, despite identical observed visitation data. Importantly, these effects extended beyond changes in the focal species itself; modifying the resource definition of a single highly connected plant species was sufficient to alter inferred interaction strengths and species classifications across the wider community. At the network level, differences in inferred pollinator preference patterns among resource definitions were detected, although the magnitude of these effects varied among field sites. Together, these findings highlight that floral resource definitions represent ecological assumptions within null model analyses rather than simple methodological choices, with consequences for how species importance and interaction patterns are interpreted in plant‐pollinator communities.

### 4.1 Effects of Floral Resource Definitions on Null Model Inference

Our results suggest that the effect of floral resource definition on null model outputs arises because estimates of floral abundance determine the expected distribution of insect visitation across plant species (Vaughan et al. 2017). When hogweed abundance was quantified by individual flowers, its estimated availability was substantially higher; this inflates the expected visitation frequency under the null model, making observed visits less likely to exceed null expectations. In contrast, defining resources at the floral unit or umbel level reduced estimated availability, increasing the standardised effect size (SES) values and the probability of hogweed being classified as a potential keystone plant.

However, these effects were not restricted to the focal species. Because preference‐based null models estimate expected interactions relative to the availability of all resources in the community, changing the abundance estimate of one highly connected species inevitably shifts the relative resource landscape against which all other interactions are evaluated. This explains why altering the resource metric of a single plant can change the inferred interaction strengths and classifications of the other co‐flowering species, even when the underlying visitation data remain completely unchanged. Ultimately, these shifts highlight the importance of explicitly defining floral resource units in preference‐based null models, particularly for species with highly aggregated floral structures, because inferred roles such as ecological hubs may partly depend on the scale at which floral resources are quantified.

### 4.2 Defining Biologically Meaningful Floral Resource Units

While individual flower counts offer a simple measure of abundance, they fail to accurately represent resource availability in species with aggregated floral structures. This variation can influence our understanding of pollinator foraging behaviour and preferences, as insect visitors assess rewards at varying spatial scales depending on floral density and display structure (Burdon et al. 2020; Campbell et al. 2012; Rosas‐Guerrero et al. 2014). This quantification challenge is widespread among plants with compound or hierarchical inflorescences, particularly within the Apiaceae and Asteraceae families, where defining a single ‘resource unit’ remains problematic. Since these species often occupy highly connected positions in plant‐pollinator networks (Bain et al. 2022; Kilian et al. 2023; Peer et al. 2026), any misrepresentation of their floral availability risks distorting our understanding of the wider network structure.

More direct measurements of floral rewards, such as nectar and pollen availability, may provide closer estimates of the resources available to pollinators (Ballantyne et al. 2015; Willmer et al. 2017; Barberis et al. 2024). However, these measurements are themselves dynamic rather than fixed properties of plant species. Nectar and pollen availability can vary with environmental conditions (Descamps et al. 2018; Takkis et al. 2018), floral display size (Biernaskie and Cartar 2004), and environmental pollutants such as heavy metals, which can compromise nectar quality, pollen viability, and pollinator foraging behaviour (Musah 2025). Furthermore, collecting detailed reward measurements across entire communities remains challenging at the spatial and temporal scales required for network studies (Hemingway et al. 2024). As a result, flower counts are likely to remain widely used proxies for floral resource availability.

Given the influence of floral resource definitions observed in this study, consistency and transparency in floral abundance measurements are essential when constructing and comparing plant‐pollinator networks. Researchers should clearly report the resource units used and consider whether these units reflect the ecological scale relevant to the study system and focal pollinators.

### 4.3 Implications for Identifying Keystone Species in Ecological Networks

Our results show that the identification of potential keystone plant species in plant‐pollinator networks can depend on how floral resources are defined. This suggests that network derived keystone classifications should be interpreted in relation to the methodological assumptions used to construct the network and associated null models.

These findings highlight that keystone identification is context‐dependent, with resource quantification representing one source of variation influencing ecological inference. More broadly, previous studies have shown that some patterns of specialisation or changes in partner choice can disappear once sampling effects and encounter frequencies are taken into account, indicating that some inferred patterns may partly reflect analytical assumptions rather than biological processes alone (Vázquez and Aizen 2003; Macleod et al. 2016). Thus, species importance inferred from network analyses should be interpreted in relation to how interactions and resource availability are represented. Similar methodological sensitivity has been reported in other ecological networks. For example, keystone taxa identification through network based methods also exhibit context‐dependent ecological relevance in microbial ecology (Banerjee et al. 2018), and differences in sampling methods have been shown to influence null model analyses in predator‐prey networks (Cuff et al. 2024). Experimental studies that manipulate floral availability could further test whether species identified as important through network analyses produce measurable effects on pollinator visitation patterns and community structure (Brosi and Briggs 2013; Biella et al. 2019). Although these systems differ substantially, they illustrate a broader principle that methodological choices can influence how species roles are inferred from ecological network analyses.

### 4.4 Limitation and Future Directions

While this study provides clear evidence of methodological sensitivity, certain limitations inherent to its spatial and taxonomic scale should be acknowledged. First, our empirical focus was restricted to a single plant species ( *Heracleum sphondylium* ) over one field season, highlighting the need to test whether these observed patterns remain across different floral structures. Second, although the study was conducted across three distinct field sites, these locations represent varying community contexts rather than formally replicated habitat types. Our results should therefore be interpreted as evidence that floral resource definition can influence null model inference across contrasting field sites, rather than as a formal comparison among different habitats.

To build upon these insights, future work should evaluate the sensitivity of network inferences across a broader range of plant taxa, floral architectures, and pollinator communities. This would help determine when individual flowers, floral units, or larger inflorescences provide the most biologically meaningful representation of floral resource availability.

## 5 Conclusion

In conclusion, this study shows that how floral resource availability is defined can influence ecological inference from plant‐pollinator null models. Using hogweed as a focal case, we found that alternative resource definitions altered its classification as a potential keystone plant, affected the inferred importance of other plant species, and changed broader patterns of inferred pollinator preference at some sites. These results highlight that floral abundance is not simply a descriptive input, but a key component of the null expectation against which visitation is evaluated. Explicitly defining and reporting floral resource units is therefore essential when using plant‐pollinator networks to infer species importance, especially for plants with compound or hierarchical floral structures. More broadly, floral resource definitions should be treated as explicit ecological assumptions rather than neutral methodological choices when interpreting plant‐pollinator network structure.

## Author Contributions

**Han Yan:** conceptualization (lead), data curation (lead), formal analysis (lead), funding acquisition (lead), methodology (lead), project administration (lead), visualization (lead), writing – original draft (lead). **Ian P. Vaughan:** formal analysis (supporting), methodology (supporting), software (supporting), supervision (equal), visualization (supporting), writing – review and editing (supporting). **Jane Memmott:** conceptualization (supporting), funding acquisition (lead), investigation (supporting), methodology (supporting), project administration (supporting), resources (supporting), supervision (lead), writing – review and editing (supporting).

## Funding

This study was funded by The Liv Sidse Jansen Memorial Foundation.

## Ethics Statement

The authors have nothing to report.

## Consent

The authors have nothing to report.

## Conflicts of Interest

The authors declare no conflicts of interest.

## Data Availability

All raw data, processed data, and R scripts supporting the findings of this study are available in Zenodo: https://doi.org/10.5281/zenodo.18483480. Code Availability: The analytical workflow was based on publicly available R functions from the packages econullnetr (Vaughan et al. 2017) and vegan (Oksanen et al. 2022), which were adapted to this study for constructing replicate null model networks, calculating standardised effect sizes (SES), and conducting NMDS and ANOVA analyses.
